# Clients and clinician satisfaction with laboratory services at selected government hospitals in eastern Ethiopia

**DOI:** 10.1186/1756-0500-6-15

**Published:** 2013-01-16

**Authors:** Zelalem Teklemariam, Abiyu Mekonnen, Haji Kedir, Getachew Kabew

**Affiliations:** 1 Department of Medical Laboratory Sciences, Haramaya University, College of Health and Medical Sciences, Dire Dawa, Ethiopia; 2 Department of Public Health, Haramaya University, College of Health and Medical Sciences, Dire Dawa, Ethiopia

**Keywords:** Laboratory services, Satisfaction, Clinical services providers, Patients, Eastern Ethiopia

## Abstract

**Background:**

In clinical laboratory service, patients and clinical service providers are the primary focus of survey of satisfaction in many countries. The objective of the study was to assess clients’ and clinicians’ satisfaction with laboratory services at selected government hospitals in eastern Ethiopia from May to June, 2010.

**Findings:**

A cross sectional study was conducted at Dil Chora, Jugal, Hiwot Fana and Bisidimo hospitals. Data were collected from 429 patients and 54 clinical service providers. A statistical analysis was conducted using Likert Scale and SPSS Version 16 software. Most of the patients (87.6%) were satisfied with the laboratory services. The lowest [2.48 ± 1.39] and highest [4.27 ± 0.83] rate satisfaction were on cleanness of latrine to collect specimens and availability of laboratory staff on working hours respectively. The extent of the patients’ satisfaction was different among the study hospitals (P-value < 0.05). Most of the clinical services providers (80%) were also satisfied with the laboratory services. The lowest [3.02 ± 1.36] and highest [3.78 ± 1.03] rate of satisfaction were found on critical value notification and timely test results for HIV/AIDS patients care respectively.

**Conclusion:**

The overall degree of customers’ satisfaction with laboratory services was high. But there were some services such as the cleanness of latrines, information given during specimen collection outside laboratory and critical value notification which need attention. Therefore, the hospital administrations and the laboratory departments should work harder and closely to solve the identified problems. Further study with a larger sample size and more factors is recommended.

## Findings

### Background

Clinical laboratories are part of the health institution team which produces important information for the patients’ care [1,2]. Laboratory services are given in all health institutions, except in health posts [3].

The problems related to clinical laboratory are aggravated particularly at peripheral level due to lack of properly designed laboratory rooms, shortage of short term and long term training for laboratory staff, lack of water and electricity, shortage of equipment and supplies, absence of effective maintenance and spare parts and lack of follow-up and supervision [3-5].

The satisfaction of customers is measured to identify problems and resolve them [6-8]. It is also an important and useful quality improvement tool for clinical laboratory, health care organizations, and business in general. Most clinical laboratories in the United States are required to assess their customers’ satisfaction in order to maintain their accreditations [9]. Some previous studies have assessed the laboratories in Ethiopia [3,4,10]. The findings of them revealed that there were shortage of manpower, equipment, chemicals and other supplies, absence of a quality assurance program network and problems in maintenance of equipment. However, customer’s satisfaction by laboratory services has not yet exhaustively studied in Ethiopia. Therefore, this study assessed clients’ and clinicians’ satisfaction with laboratory services at selected government hospitals in eastern Ethiopia.

### Methods

#### Study area, period and design

A cross sectional study was conducted in Dil Chora, Hiwot Fana, Jugal and Bisidimo Hospitals, Ethiopia, from May to June , 2010. Dil Chora Hospital is found in Dire Dawa Administrative Council, which is 500km away from Addis Ababa. Hiwot Fana and Jugal Hospitals are found in Harari National Regional State, which is 511Km away from Addis Ababa. Bisidimo Hospital is found in eastern Hararghe , Babile Wareda, which is 20 km away from Harar. Dil Chora , Hiwot Fana , Jugal and Bisidimo hospital had four, three, two and two places for laboratory services respectively. The laboratory services provided by these hospitals include: stool, urine, clinical chemistry, hematology, serology, CD_4_ count, bacteriology (gram’s and acid fast stain) and others. Dil Chora, Hiwot Fana, Jugal and Bisidimo hospital laboratories had average patient flow of 3000, 2430, 2010 and 1260 per month, respectively.

#### Study subject, sample size and sampling procedure

All the clinical service providers (physicians and public health officers) who were on duty during the study period were included in this study. In addition, randomly selected patients who came to each hospital laboratories were interviewed.

The sample size for the patients was determined by using P = 0.5, margin of error 0.05 and a non respondent rate of 10%. Therefore 429 patients were included. Then it was distributed proportionally to each hospitals based on the average patient flow per month. A total of 148, 120, 99 and 62 patients were included from Dil chora, Hiwot Fana, Jugal and Bisidemo hospitals, respectively. While 15, 13, 14 and 12 clinical service providers were included from Hiwot Fana , Jugal , Dil Chora and Bisedimo hospital, respectively.

#### Methods of data collection and measurement

##### A. Patients

Data were collected by face-to-face interview using structured questionnaire. The patients who had finished their laboratory examinations and returned to Out Patient Department (OPD) and willing to participate in this study were interviewed. The questionnaire contained the socio-demographic characteristic, length of time to take results, the availability of laboratory staff on working hours, location and cleanness of latrine and other.

##### B. Clinical service providers

A self administered questionnaire was given to physicians and public health officers and then collected at the end of each day. The questionnaire contained the socio-demographic characteristic, courtesy of the laboratory staff, critical value notification, courier service, reliability of test results, provision of a timely test results for HIV/AIDS patients care and others.

#### Statistical analysis

Data entry and analysis was made using SPSS 16 software. A 5 point Likert Scale rating of Poor (1-point) ,Fair (2-points), good (3-points), very good (4-points) and excellent (5point) were used. Association of the variables was checked by using Chi-square test. P-value < 0.05 was considered as statistically significant. Univariate and multivariate logistic regression was employed to determine the possible socio-demographic characteristics associated with the level of satisfaction. A variable with P < 0.2 in univariate analysis was included in multivariate analysis. Poor and fair responses were considered as dissatisfied, whereas good, very good and excellent were considered as satisfied. Clients with neutral rating responses were excluded. The percentage satisfaction or dissatisfaction was calculated by dividing the number of satisfied or dissatisfied responses by the total number respondents excluding neutral response ratings, respectively.

The overall rate of satisfaction by Likert scale was calculated as (No. of excellent rating x5) + (No. of very good rating x4) + ( No. of good rating x3) + ( No. of fair rating x2) + ( No. of poor rating x1) divided by the total number of ratings (1–5) for the specific laboratory service. While the percentage of excellent, very good, good, fair or poor rating was calculated by dividing the number of excellent, very good, good, fair or poor rating by the total number of ratings (1–5) for specific laboratory service, respectively.

##### Ethical consideration

Ethical clearance was obtained from Institutional Research and Ethical Committee of Haramaya University. Questionnaires were distributed after getting informed and signed consent from the respondents.

### Results

#### Socio-demographic characteristics of the participants

A total of 429 patients and 54 clinical service providers participated in this study. Among the study subjects, 53.1% of the patients and 31.5% of the clinical services providers were female. The mean ± SD of the patients and clinical services providers’ age were 34 ± 13.8 and 30 ± 9.2 years, respectively.

The distribution of patient participants with regard to educational status and residence revealed that 31.9% were illiterate (unable to read) and 58.7% were urban residents. Majority, (40.8%), of patient respondents were also in the age group of 18–27 years, 31.2% were farmer and 62.5% were married (Table 1).

**Table 1:** **Distribution of socio** –**demographic characteristics of patient respondents by their percentage of level of satisfaction at selected hospitals in eastern part of Ethiopia**, **2010**

**Variables**	**Dissatisfied**	**Satisfied**	**Neutral**	**Total**	**X**^**2**^	**df**	**P**-**value**
	**No** (%)	**No** (%)		**No** (%)			
**Gender**							
Male	28(6.7)	168( 40.1)	5	201(46.9)	1.19	1	0.28
Female	24(5.7)	199(47.5)	5	228(54.1)			
**Age**							
18-27	29(6.9)	141(33.7)	5	175(40.8)	6.02	4	0.19
28-37	9(2.1)	102(24.3)	1	112(26.1)			
38-47	8(1.9)	67(16.0)	2	77(17.9)			
48-57	3(0.7)	25(6.0)	1	29(6.8)			
≥ 58	3(0.7)	32(7.6)	1	36(8.4)			
**Marital status**							
Single	15(3.6)	99(23.6)	2	116(27.0)			
Married	37(8.8)	223(53.2)	8	268(62.5)			
Divorced	0	18(4.3)	0	18(4.2)			
Widowed	0	27(6.4)	0	27(6.3)			
**Educational status**							
Unable to read and write	17(4.0)	116(27.7)	4	137(31.9)			
Able to read and write	1 (0.2)	13 (3.1)	0	14(3.3)	5.1	6	0.53
1-4	4 (1.0)	41 (9.8)	1	46(10.7)			
5-8	10(2.4)	84 (20.0)	3	97(22.6)			
9-10	9 (2.1)	36 (8.6)	0	45(10.5)			
11-12	6 (1.4)	27 (6.4)	1	34(7.9)			
12^+^	5 (1.2)	50 (11.9)	1	56(13.1)			
**Occupation**							
Government employee	8(1.9)	54(12.9)	0	62(14.5)	1.70	6	0.95
Merchant	7(1.7)	46(11.0)	1	54(12.0)			
Farmer	19(4.5)	110(26.3)	5	134(31.2)			
NGO’s	1(0.2)	8(1.9)	0	9(2.1)			
Retired	4(1.0)	36(8.6)	2	42(9.8)			
Daily laborer	8(1.9)	60(14.3)	1	69(16.1)			
Student	5(1.2)	53(12.6)	1	59(13.8)			
**Residence**							
Urban	25 (6.0)	214 (51.0)	7	250(58.7)	2.4	1	0.12
Rural	27(6.4)	149 (35.6)	3	179(41.3)			
**No**. **times of patients visit laboratory in this year**							
1times	11( 2.6)	59 (14.1)	3	73(17.0)	2.9	3	0.41
2times	10 (2.4)	55( 13.1)	1	66(15.4)			
3times	25( 6.0)	222 (53)	5	252(58.7)			
More than 3 times	6 (1.4)	31(7.4)	1	38(8.9)			
**Selected Hospitals name**							
Bisidemo	6(1.4)	56(13.4)	0	62(14.5)			
Jugal	18 (4.3)	77(18.4)	4	99(23.1)			
Hiwot Fana	27(6.4)	87(20.8)	6	120(28)			
Dil Chora	1(0.2)	147(35.1)	0	148(34.5)	36.2	3	0.00

#### Degree of satisfaction of the patients

Most of the patients (87.6%) were satisfied with the laboratory services. Rate of satisfaction was statistically different by the study hospitals (P-value < 0.05). More females than males, urban dwellers than rural residents, illiterates than literates, and clients who visited a laboratory three times were than those who visited less than three times were satisfied. Similarly rate of satisfaction is higher among clients in age group of 18–27 years and farmers compared to other occupation. However the differences were not statistically significant for all socio-demographic characteristics described above (P > 0.05) (Tables 1 and 2).

**Table 2:** **Univariate and Multivariate analysis to assess predicator socio**- **demographic variables with the satisfaction of patients at selected hospitals in eastern part of Ethiopia**, **2010**

**Variables**	**Crude OR** (**95**% **CI**)	**P**-**value**	**Adjusted OR** (**95**% **CI**)	**P**-**value**
**Gender**				
Female	1.00			
Male	1.38(0.77,2.48)	0.28		
**Age**				
18-27	1.00			
28-37	2.19(0.62,7.65)	0.22		
38-47	0.94(0.24.3.69)	0.93		
48-57	1.27(032.5.12)	0.73		
≥ 58	1.28(024,6.89)	0.77		
**Educational status**				
illiterate	1.00			
Literate	1.05(0.57,1.95)	0.88		
**Occupation**				
NGO’s	1.00			
Retired	0.72(0.86,6.12)	0.77		
Merchant	0.64(0.21,2.01)	0.45		
Student	0.88(0.35,2.24)	0.79		
Government employee	0.55(0.19,1.54)	0.25		
Daily laborer	0.86(0.35,2.08)	0.74		
Farmer	0.77(0.32,1.87)	0.57		
**Residence**				
Urban	1.00		1.00	
Rural	0.63(0.35,1.13)	0.12	0.74(0.38,1.44)	0.37
**No**. **times of patients visit laboratory in this year**				
1 -2times	1.00		1	
More than 3 times	1.50(0.83,2.73	0.18	1.49(0.74,3.00)	0.27
**Hospital name**				
Bisidemo	1.00			
Jugal	15.75(1.85,133.77)	0.01	12.88(1.45,114.4)	0.02
Hiwot Fana	34.36(4.50,262.29)	0.00	29.82(3.85,231.03)	0.01
Dil Chora	45.62(6.09,341.66)	0.00	44(5.81,332.81)	0.00

#### Univariate and Multivariate analysis

In univariate analysis, only selected hospital, number time patients visit laboratory this year and residence was significantly associated with the percentage of patient’s satisfaction (P < 0.2). However, in multivariate analysis patients visiting Dil Chora hospital were the most satisfied group (AOR = 44; CI: 5.81- 332.81) (Table 2).

In Likert Scale, the overall mean rate of satisfaction of patients by the laboratory services was 3.45 ± 0.85. The mean rate of satisfaction for different aspects of laboratory services ranged from 2.48 ± 1.39 to 4.27 ± 0.83. Cleanness of latrine and information given for specimen collection outside the laboratory room were given the lowest mean rating of 2.48 ± 1.39 and 2.67 ± 1.31 respectively. Higher mean rating of satisfaction was obtained for availability of laboratory staff on working hours (4.27 ± 0.83) and willingness to conduct laboratory investigation (4.12 ± 1.62) as shown in Table 3.

**Table 3:** **Rate of patients**’ **satisfaction by different measuring item of laboratory services at selected study hospitals at eastern part of Ethiopia**, **2010**

**Variable**	**Poor**	**Fair**	**Good**	**Very good**	**Excellent**	**Neutral**	**Mean rating**	**Satisfied percentage**
	**No**(%)	**No**(%)	**No**(%)	**No**(%)	**No**(%)			
Willingness to conduct laboratory investigation	3(0.7)	7(1.6)	59(13.8)	224(52.5)	134(31.4)	1	4.12 ± 0.75	97.2
Location of the laboratory in the hospital	28(6.6)	19(4.5)	7818.4)	227(53.5)	72 (17)	5	3.70 ± 1.02	88.2
Cleanness and attractiveness of the laboratory room	8(1.9)	11(2.6)	100(23.9)	201(48.1)	98(23.4)	9	3.89 ± 0.86	95.5
Availability of laboratory staff on working hours	6(1.4)	6(1.4)	51(12.1)	167(39.5)	193(45.6)	3	4.27 ± 0.83	97.2
courtesy of laboratory staff	12(2.8)	17(4.0)	77(18.2)	233(55.2)	83(19.7)	5	3.85 ± 0.88	93.1
Conduct of laboratory staff during specimen collection like blood	11(3.2)	14(4.1)	68(20.1)	198(58.4)	48(14.2)	86	3.76 ± 0.86	92.6
Information given during specimen collection outside the laboratory room	129(34.0)	31(8.2)	70(18.5)	133(35.1)	16(4.2)	49	2.67 ± 1.36	57.8
Location of latrine to collect specimens	70(19.3)	30(8.3)	77(21.2)	170(46.8)	16(4.4)	66	3.09 ± 1.22	72.5
Cleanness of latrine to collect specimens	146(40.0)	43(11.8)	46(12.6)	116(31.8)	14(3.8)	63	2.48 ± 1.39	75.6
Length of time to take results back to physician	37(8.8)	48(11.4)	118(28.0)	151(35.9)	67(15.9)	8	3.39 ± 1.15	80.0
Perception about quality of laboratory results	10(2.4)	34(8.0)	94(22.2)	218(51.5)	67(15.8)	6	3.70 ± 0.91	89.6
Language of laboratory staff used to communicate	14(3.3)	17(4.0)	60(14.0)	176(41.0)	162(37.8)	0	4.06 ± 0.98	92.8
*General satisfaction by the hospital laboratory	15(3.6)	37(8.8)	132(31.5)	216(51.6)	19(4.5)	10	3.45 ± 0.85	87.6

* It was calculated from single question.

**Figure 1:**
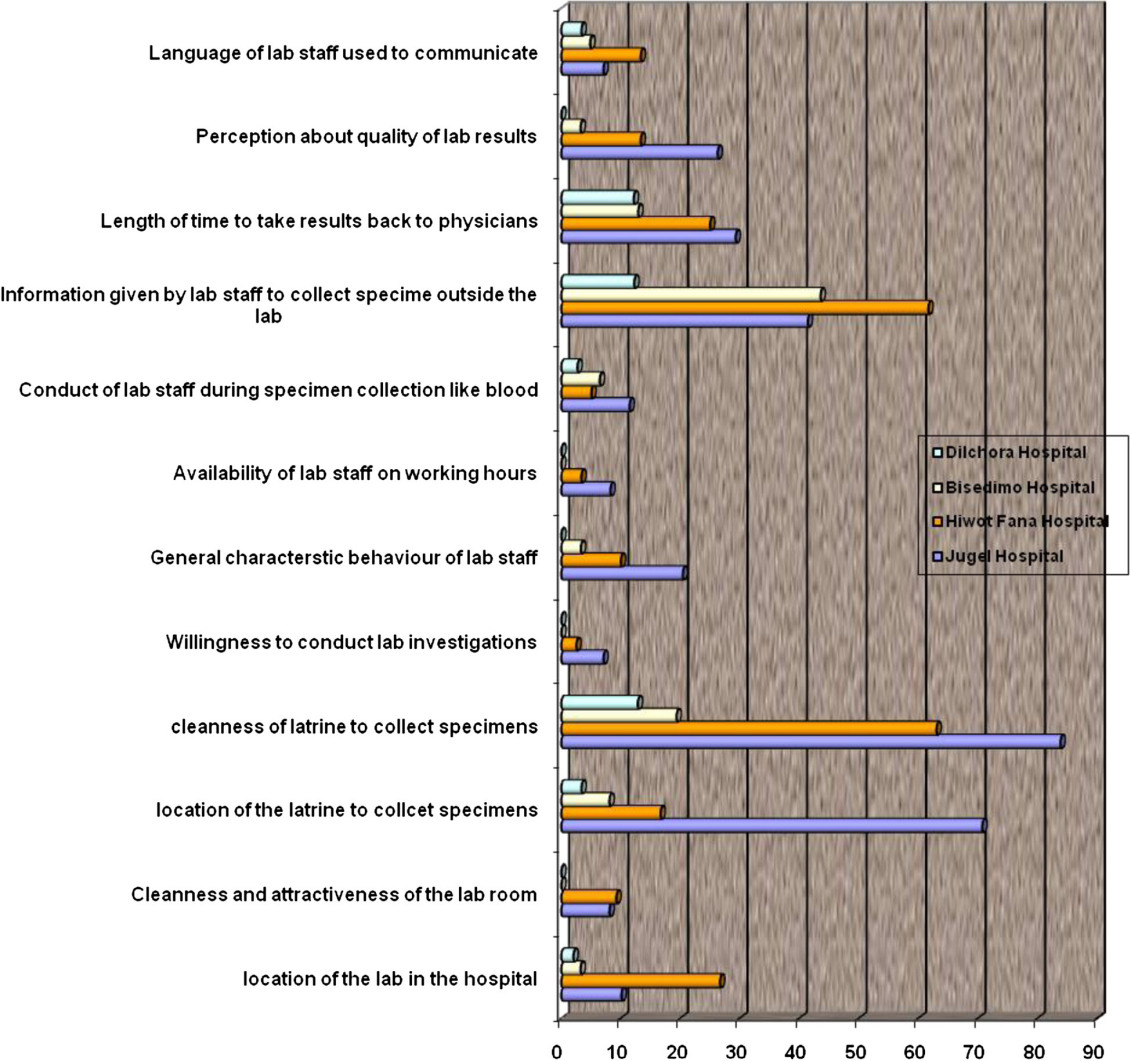
Level of dissatisfaction of patients by different measuring items of laboratory services at selected hospitals in eastern part of Ethiopia, 2010.

The highest dissatisfaction was observed by cleanness and location of the latrine to collect specimens in Jugal and information given by the laboratory staffs to collect specimens in Hiwot Fana Hospital (Figure 1).

#### Degree of satisfaction of clinical service providers

The overall percentage of satisfied clinical services providers by the laboratory services was 80.0%. Based on responses from Likert Scale, overall mean rate of satisfaction was 3.49 ± 1.27. The lowest [3.02 ± 1.36] and the highest [3.78 ± 1.03] rate of satisfaction were found for critical value notification and timely test results for HIV/AIDS patients care, respectively (Table 4).

**Table 4:** Rate and percentage of satisfaction of clinical service providers by different measuring items of Laboratory services at selected hospitals in eastern part of Ethiopia, 2010

**Variable**	**Poor**	**Fair**	**Good**	**Very good**	**Excellent**	**Neutral**	**Mean rating**	**Satisfied percentage**
	**No**(%)	**No**(%)	**No**(%)	**No**(%)	**No**(%)			
Availability of laboratory staff on working hours	1(2)	4(8)	19(38)	13(26)	13(26)	4	3.66 ± 1.02	90.0
Courtesy of laboratory staff	1(2)	8(16)	23 (46)	10(20.0)	8(16)	4	3.32 ± 1.00	82.0
Adequacy of test menu on laboratory request format	1(2.0)	5(10.0)	14(28.0)	20(40.0)	10(20.0)	4	3.66 ± 0.98	88.0
Critical value notification of the laboratory staff	8(16.3)	11(22.4)	11(22.5)	10(20.4)	9(18.4)	5	3.02 ± 1.36	61.2
Satisfaction by departmentalization of the hospital laboratory	1(2)	4(8.1)	17(34.7)	20(40.8)	7(14.2)	5	3.57 ± 0.91	89.8
Specimen collection of the laboratory staff	7(13.5)	3(5.7)	13(25.0)	19(36.5)	10(19.2)	4	3.42 ± 1.26	84.0
Courier service of the laboratory	4(8.1)	6(12.2)	20(40.8)	8(16.3)	11(22.4)	5	3.33 ± 1.10	79.6
Quality/reliability of laboratory test results	2(4.1)	8(16.3)	15(30.6)	20(40.8)	4(8.2)	5	3.33 ± 0.99	79.6
Reporting of complete test results	2(4.0)	10(20.0)	15(30.0)	20(40.0)	3(6.0)	4	3.27 ± 0.97	76.0
Laboratory staff in avoiding missing of laboratory results	2(3.9)	6(11.7)	25(49.1)	10(19.6)	8(16)	3	3.31 ± 1.00	84.3
Timely test results for HIV/AIDS patients care	-	8(16.3)	8(16.3)	20(40.8)	13(26.5)	5	3.78 ± 1.03	83.7
Getting urgent results on time	1(2.0)	6(12.0)	9(18.0)	28(56.0)	6(12.0)	4	3.64 ± 0.92	86.0
STAT test results on time	2(4.0)	4(8.0)	14(28.0)	20(40.0)	10(20.0)	4	3.64 ± 1.03	88.0
*General satisfaction by the hospital laboratory	4(8.0)	6(12.0)	15(30.0)	10(20.0)	15(30.0)	4	3.49 ± 1.27	80.0

* It was calculated from single question.

### Discussion

The overall mean rating of satisfaction by patients was 3.45 ± 0.85, which is lower than a study conducted in a student run medical clinic, in which mean satisfaction of 4.41 has been reported [11]. This could be due to differences in the setups of the laboratory. The percentage of satisfaction was similar to a study on laboratory service in antiretroviral therapy clinics in Addis Ababa, Ethiopia [12]. But it was higher than another study carried in Eastern Ethiopia [13]. This might be due to the large number of trained medical laboratory professionals deploying in the services. In addition, it might be due to availabilities of different laboratory services.

The level of satisfaction of the patients was not statistically different by educational status and residence, and which is similar to the finding by Abdosh [13]. However it was statistically different by the study hospitals. This could be due to the basic difference in infrastructure, roles of the respective hospital administrations, financial and human resources.

Higher rate of patient’s satisfaction was on the availability of laboratory staff on working hours and willingness to conduct laboratory investigation. Lower rate was obtained on provision of appropriate information to patients. Similarly, lack of explanation about the system is one of the dissatisfying factors in study from Tanzania [7]. The findings could be due to low attention given for the activities outside the laboratory room where tests are conducted, work overload or other reasons. These imply the importance of all laboratory environments on client satisfaction. Cleanness of latrine and location of latrine, i.e. the difficulty in searching the location of the latrine to provide specimens like stool and urine, were also reported as the lowest rating of patients’ satisfaction, which is consistent with other studies in Ethiopia [12,13]. This might be due to laboratory personnel might not participate at time of reviewing the design of requirement of laboratory before construction.

The overall mean rate of satisfaction among clinical service providers was 3.49 ± 1.27. The highest rate of satisfaction was observed on timely test results for HIV/AIDS patients care. The lowest rate of satisfaction was observed on critical value notification and on reporting of complete test results. This is almost similar with the finding from Tanzania [14], where personnel were dissatisfied with incomplete results. This implies the need for improving laboratory services in terms quality, which could be by quality assurance officer or head of a laboratory.

#### Limitation

This study did not assess the laboratory personnel awareness about customers’ need and set up of each hospital laboratory. In addition, the sample size allocated to each hospital is not large enough to assess the significance level of satisfaction by different factors due to low power.

### Conclusions

The overall degree of customers’ satisfaction with the laboratory services was high. But there were some services such as the cleanness and location of latrines, information given for specimen collection outside laboratory and critical value notification which need attention. Therefore, the hospital administrations and the laboratory departments should work harder and closely to solve the identified problems. In addition, each hospital should monitor the quality of their laboratory services, use client feedbacks and assess customer satisfaction. Further study with a larger sample size and more factors is recommended.

## Competing interests

All the authors declare that they have no conflict of interest associated with the publication of this manuscript.

## Authors’ contributions

AM conceived and designed the study. AM and ZT contributed significantly in reviewing the proposal, data collection, analysis, interpreted the data and drafted the manuscript. HK significantly contributed in data collection, analysis, interpreted the data, and reviewed the manuscript. GK significantly involved in data collection and has reviewed the manuscript. All authors read and approved the final manuscript.
